# Dr. Edward George Whittle

**Published:** 1909-05-22

**Authors:** 


The death of Dr. Edward George Whittle, M.D.(Lond.), F.R.C.S.(Eng.), who for the past 33 years had resided at Brighton, is announced to have taken place at Las Palmas, at the age of 59. Dr. Whittle was one of the leading practitioners of Brighton until his retirement from active work a few years ago. After studying medicine at University College Hospital, he qualified in 1873, and subsequently obtained the higher degree and diploma of the University of London and the College of Surgeons. He was President of the Brighton Medico-chirurgical Society in 1891, and for some years he was honorary physician to the Brighton and Hove Dispensary and surgeon to the Royal Alexandra Hospital for Children.

